# Twenty Years of *Acanthamoeba* Diagnostics in Austria

**DOI:** 10.1111/jeu.12149

**Published:** 2014-09-25

**Authors:** Julia Walochnik, Ute Scheikl, Eva-Maria Haller-Schober

**Affiliations:** a Molecular Parasitology, Institute of Specific Prophylaxis and Tropical Medicine, Centre for Pathophysiology, Infectiology and Immunology, Medical University of ViennaVienna, Austria; b Department of Ophthalmology, Medical University of GrazGraz, Austria

**Keywords:** characterisation, contact lens, infection, protozoa, SSU, typing

## Abstract

Acanthamoebae are the causative agents of an often seriously progressing keratitis (AK) occurring predominantly in contact lens wearers and can cause several disseminating infections potentially resulting in granulomatous amoebic encephalitis (GAE) in the immunocompromised host. Our institution is the Austrian reference laboratory for *Acanthamoeba* diagnostics and the aim of this study was to give an overview of proven cases of *Acanthamoeba* infections in Austria during the past 20 yr. All samples of patients with suspected AK or GAE were screened for *Acanthamoeba* spp. by culture and/or PCR and the detected amoebae were genotyped. Altogether, 154 cases of AK and three cases of GAE were diagnosed. Age of the AK patients ranged from 8 to 82 yr (mean 37.8) and 58% of the patients were female. Approximately 89% of the AK patients were contact lens wearers, almost all cases were unilateral and 19% of the patients required a keratoplasty. Age of the GAE patients ranged from 2 to 25 yr (mean 14.7), all were HIV-negative, but two were severely immunosuppressed at the time of diagnosis. The predominant genotype in the AK cases was T4, other genotypes found were T3, T5, T6, T10 and T11. The three GAE cases involved genotypes T2, T4 and T5.

ACANTHAMOEBAE are primarily free-living microorganisms that can however cause severe disease when they enter the human body (reviewed in Visvesvara 2010, 2013). *Acanthamoeba* keratitis (AK) is a very painful infection of the eye and has become of growing importance within the past decades correlating to the increasing number of contact lens (CL) wearers, but also to increased awareness (Ku et al. 2009; Lorenzo-Morales et al. 2013; Patel and Hammersmith 2008). Besides this, *Acanthamoeba* spp. are the causative agents of several disseminating infections in the immunocompromised host, potentially resulting in granulomatous amoebic encephalitis (GAE). Generally, water and air are the most probable vehicles for transmission however, the establishment of an *Acanthamoeba* infection is dependent on the size of the inoculum, the immune status of the patient and the virulence and invasiveness of the particular amoeba strain.

The first recognised case of AK occurred in 1973 in a Texas rancher (Jones et al. 1975). Nagington et al. (1974) reported two further cases of AK, in a school-teacher and a farmer, respectively, and these were the first recorded cases in the UK. In two of these three cases, infection followed a minor trauma in the eye leading to a compromised corneal barrier. In Central Europe, the first case of AK was reported in 1984 from Germany and was one of the very first cases of CL-associated AK. The patient was a 63 yr old female wearer of rigid CLs and recovered after keratoplasty (Witschel et al. 1984). Soon, the association between AK and the wearing of soft CLs was established (Moore et al. 1985) and more and more cases were reported from all over the world, including cases in non-CL wearers (reviewed in Jain et al. 2013; Lorenzo-Morales et al. 2013).

The first clearly identified human case of *Acanthamoeba* GAE occurred in an Amercian patient with Hodgkin's disease (Jager and Stamm 1972) and the first case of GAE in an AIDS patient was recorded in 1986, also in the USA (Wiley et al. 1987). Since then, several hundred GAE cases have been reported worldwide (reviewed in Marciano-Cabral and Cabral 2003; Martinez and Visvesvara 1997; Zamora et al. 2014). In 1990, it became apparent that another free-living amoeba can cause GAE, namely *Balamuthia mandrillaris*, which had been isolated from the brain of a mandrill from the San Diego zoo (Visvesvara et al. 1990) and was described as a new species in a new genus (Visvesvara et al. 1993). In 1992, the first European case of *Acanthamoeba* GAE in an AIDS patient was reported from Italy (Di Gregorio et al. 1992), and the first European case of *Balamuthia* GAE was published in 1998 in the Czech Republic (Kodet et al. 1998).

In Austria, to the best of our knowledge, there has been no case of *B. mandrillaris* infection. The first diagnosed Austrian case of AK was described in 1989, the causative agent being identified as *Acanthamoeba rhysodes* (Huber-Spitzy et al. 1989). The first recorded Austrian case of GAE occurred in 2004 (Aichelburg et al. 2008).

Our institution is the Austrian reference laboratory for *Acanthamoeba* diagnostics and the aim of this study was to give an overview of the cases of *Acanthamoeba* infections diagnosed in Austria during the past 20 yr. First samples were only investigated by culture, in 1996 an *Acanthamoeba*-specific polymerase chain reaction (PCR) was established in our laboratory and from then on, all samples from patients with suspected AK or GAE were screened for acanthamoebae by culture and PCR, and all detected amoebae were genotyped.

## Materials and methods

### Patients and samples

From 1993 to 2013, 381 samples from 282 patients with keratitis and samples from 25 patients with unclear encephalitis were investigated for *Acanthamoeba*. A majority of patients had already been under medication before the tentative diagnosis for an *Acanthamoeba* infection was made.

Generally, all samples received until 1996 were investigated only by phase contrast microscopy (Nikon Eclipse E800 microscope; Nikon Instruments, Vienna, Austria) and culture, whereas from 1996 onwards, all samples were also investigated by PCR. All liquid samples ≤ 200 μl were mixed and split into two aliquots directly upon receipt, one aliquot was transferred to a culture plate immediately and investigated daily for amoebae by phase contrast microscopy, the other aliquot was used for DNA isolation. Liquid samples > 200 μl were centrifuged at 700 *g*/7 min, resuspended in 200 μl of sterile 0.9% NaCl, split into two aliquots and processed as described above. When CLs or swabs were received, the lenses/swabs were shaken vigorously in the original solution (CL solution/sterile NaCl) and then the lens/swab was inoculated onto an agar plate and the liquid was further processed as described above. When CL cases were received, the liquid was processed as described above, but also a biofilm was swabbed from the inner surface and inoculated onto an agar plate. When cornea scrapes were received, the scrape itself was used for DNA isolation, while the transport solution (200 μl of sterile NaCl) was further processed as described above. Larger tissue samples were cut into two halves, of which one was transferred onto an agar plate and one was used for DNA isolation. In some cases, we received fixed material (swabs/CL case cell pellets or tissue samples), either in ethanol or embedded in paraffin or even as stained sections on microscopic slides. In these cases, staining (lactophenol cotton blue [LPCB] and/or immunostaining) and/or PCR were performed, and DNA was isolated using the suitable protocol for the respective material (see below).

### Culture

For culture, the respective material (liquids, CLs, corneal scrapings and biopsies) was inoculated centrally onto a nonnutrient agar (1.5%) plate covered with 100 μl of a 24 h old culture of *Escherichia coli* in bacterial broth. The plates were sealed with parafilm, incubated at 30 °C for 7 d and examined every 24 h for amoebal growth. From positive samples clonal cultures were prepared by transferring single cysts to a fresh plate. Isolates were sub-cultured every 2–4 wk. Temperature tolerances of all isolated acanthamoebae were tested by incubating sub-cultures of the respective isolates at 34 °C and 37 °C.

Isolated amoebae were investigated by phase contrast microscopy and identified at the group level (*Acanthamoeba* sp. group I-III) according to Pussard and Pons (1977), mainly based on size, cyst morphology and number of opercula. For genotyping, actively growing amoebae (∼10^6^ cells) were harvested from the culture plates as described previously (Walochnik et al. 2000) and resuspended in 100 μl of sterile 0.9% NaCl for DNA isolation.

### DNA isolation

Until 2002, whole-cell DNA was isolated by a modified UNSET (Hugo et al. 1992) procedure as described previously (Walochnik et al. 2000). In brief, tissue samples or cells suspended in 100 μl of the respective liquid (sterile 0.9% NaCl, CL solution, CSF, etc.) were disrupted by multiple freeze-thawing, further suspended in 700 μl of UNSET-lysis buffer, overlaid with an equal volume of phenol-chloroform-isoamylalcohol (PCI), and shaken gently for 5 h. The suspension was centrifuged at 3,000 *g* for 10 min, and the upper, aqueous phase was transferred to a new tube. PCI extraction was repeated twice for 10 min. Nucleic acids were precipitated by ethanol (15 min at 80 °C), pelleted at 12,000 *g* for 30 min at 4 °C, washed in 70% ethanol, air-dried and resuspended in 30 μl of sterile double-distilled water.

From 2002 onwards, whole-cell DNA was isolated from the amoebal suspensions using the QIAmp DNA mini kit (Qiagen GmbH, Germany) and following the manufacturer's protocol for the respective type of material. When larger tissue samples were received, samples were homogenised using a PeqLab homogeniser (PeqLab, Polling, Austria) prior to DNA isolation.

### Stains

Lactophenol cotton blue stain (LPCB) was performed according to the protocol of Thomas and Kuriakose (1990). Briefly, pellet suspensions were mixed with the aliquot volume of LPCB stain (Sigma-Aldrich, Vienna, Austria) and stained slides were investigated by light microscopy on a Nikon TMS. This stain is particularly well suited for *Acanthamoeba* cysts and allows the identification of the morphological group; the cyst walls appear in an intensive blue.

For immunostaining, polyclonal anti-*Acanthamoeba* antisera, produced by immunisation of a rabbit with *Acanthamoeba* whole cell antigen was used. In our lab, we have antisera against the three *Acanthamoeba* groups (I–III) available. Tissue sections were incubated with the respective anti-*Acanthamoeba* antiserum, washed, stained with fluorescein isothiocyanate linked antirabbit antibodies and counterstained with Hoechst 33258. Control stainings were performed with haematoxylin & eosin.

### Polymerase chain reactions

As a standard diagnostic *Acanthamoeba*-specific PCR (for older isolates in retrospect), we used the protocol established by Schroeder et al. (2001) amplifying a fragment of the 18S rRNA gene using the JDP1 and JDP2 primers and giving an approximately 500 bp amplicon depending on the genotype. All samples were run using 1, 3 and 6 μl whole-cell DNA respectively, in 50 μl reaction volumes. A genotype T4 *Acanthamoeba castellanii* strain (strain 1BU, ATCC PRA-105) was used as a positive control, DNA-free water as a negative control. Amplicons were visualised by ethidium-bromide (or GelRed™; VWR, Vienna, Austria) in an agarose-gel electrophoresis and the respective bands extracted from the gels with a DNA band purification kit (GE Healthcare Europe GmbH, Vienna, Austria).

For samples that had been fixed in formaldehyde, a modified PCR using the JDP1 primer from the PCR described above and the P2rev primer (Walochnik et al. 2004) and amplifying a shorter (∼300 bp) fragment of the 18S rRNA gene was performed.

### Sequencing

Sequences of the PCR amplicons were obtained by direct sequencing using the ABI PRISM® BigDye sequencing kit and a 310 ABI PRISM® automated sequencer (Applied Biosystems, Langen, Germany). Sequences were generally obtained from both strands (and in most cases from the entire 18S rRNA gene), assembled to give a consensus sequence and compared to sequences of *Acanthamoeba* reference strains. Genotypes were assessed by multiple alignments with all at that time available genotypes using CLUSTAL X (Thompson et al. 1997) and the GeneDoc sequence editor (Nicholas et al. 1997) with the model assumption of a < 5% sequence dissimilarity within one genotype as established by Gast et al. (1996).

## Results

The number of samples sent in for *Acanthamoeba* diagnostics emerged from three in 1993 to currently approximately 100 samples per year. The year with the highest number of samples was 2007, with 111 samples. The number of diagnosed cases of *Acanthamoeba* infections emerged from 1 to 2 in the first years, to currently approximately 10 per year.

### AK cases

**Figure 1:**
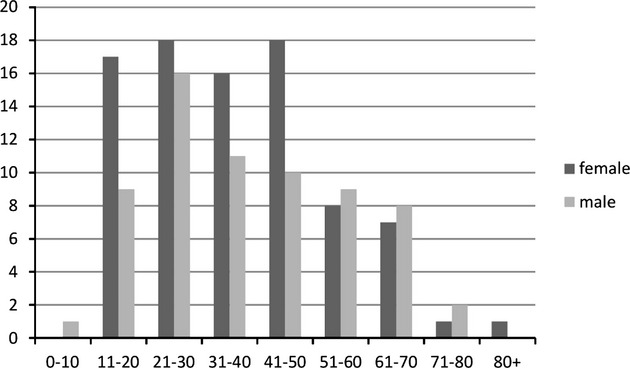
Age and sex distribution of AK patients in Austria (1993–2013).

**Figure 2:**
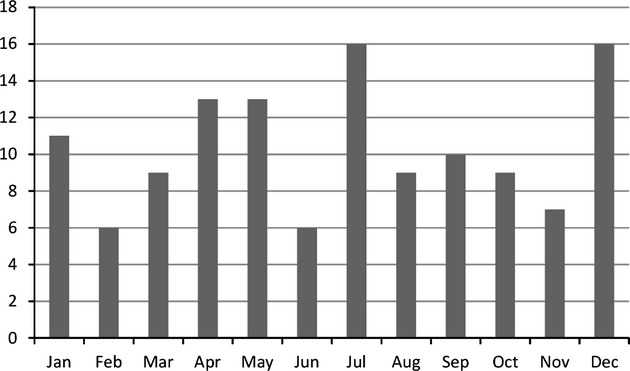
AK cases per month over the period 1993–2013.

Altogether, *Acanthamoeba* diagnosis was positive in 154 patients. Age of the AK patients ranged from 8 to 82 yr (mean age 37.8) and 58% of the patients were female (Fig.1). There was no clear correlation with seasonality, the 2 mo with the most cases were July and December, while the fewest cases occurred in February and June (Fig.2). However, these dates only approximately reflect the onset of symptoms, and, of course, not at all the time point of infection, which could have been several weeks, in rare cases possibly even months before. In approximately half of the cases, background information on the respective case was available. Of these, 89% were CL wearers, with three exceptions all cases were unilateral and 19% of these patients required a keratoplasty.

The majority of cases had a prolonged progression, patients were often not without symptoms before several months after onset of treatment. In some cases, the infection was viable for 1 yr or even longer and there were several cases of severe AK, all of them in CL wearers. In a 66 yr old female CL wearer, the infection re-emerged 6 mo after keratoplasty and the patient had to undergo a second keratoplasty. A 55 yr old male CL wearer had to undergo four surgeries for clearance of the acanthamoebae from his eye, interestingly in this case after the first unsuccessful keratoplasty also superficial swab samples from the conjunctiva and also from the mouth were positive for the same strain (100% sequence identity over the entire 18S rRNA gene). In one case, a 41 yr old male CL wearer was diagnosed with severe AK and his 14-yr old daughter, also a CL wearer, had the same *Acanthamoeba* strain (100% sequence identity over the entire 18S rRNA gene) in her CL case, but never developed disease.

**Figure 3:**
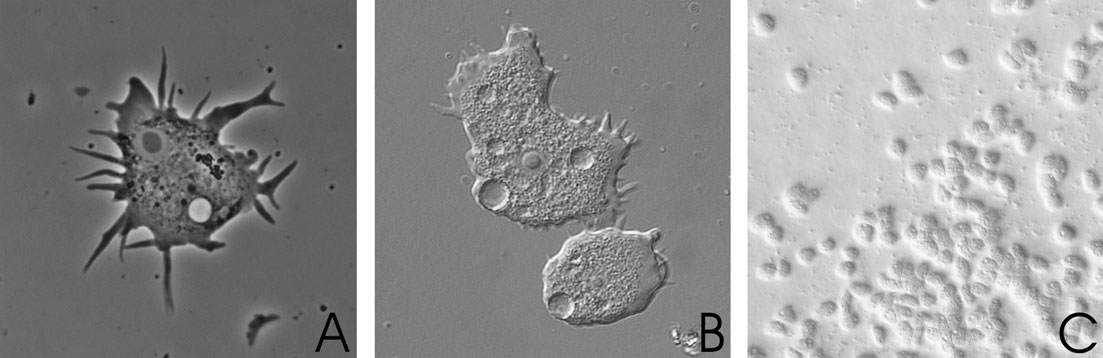
*Acanthamoeba* trophozoites. A. In phase contrast microscopy (magnification: X1,000), B. in interference contrast microscopy (magnification: X1,000) and C. On agar plate 48 h after inoculation of clinical sample in inverted phase contrast microscopy (magnification: X200).

**Figure 4:**
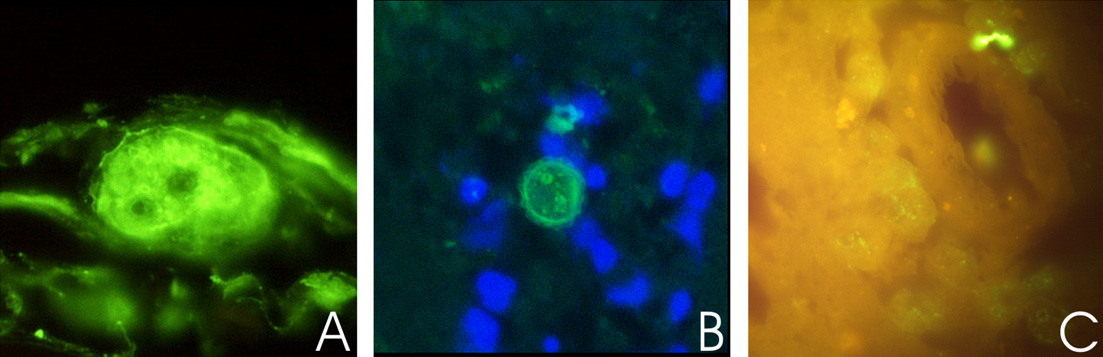
*Acanthamoeba* immunostaining. A. Trophozoite with characteristic nucleus (large central nucleolus) in superficial tissue layers (magnification: X1,000), B. Mature double-walled cyst in cornea tissue (magnification: X400) and C. Immature cysts in brain tissue around vessel (magnification: X400).

In severe AK cases, amoebae were usually already visible when the samples were screened by microscopy directly upon inoculation onto the agar plates and plates were entirely covered with amoebae after 24–48 h (Fig.3). However, particularly samples from patients that had already received antibiotic or antimycotic treatment needed rather several days (up to 1 wk) before amoebae were visible on the agar plates, or cultures remained negative altogether. This was particularly seen in follow-up samples from patients who had already been under antiamoebic treatment for some time. Sensitivity of culture reached 85% in initial samples (the first sample received from the respective patient) and went down to 10% in follow-up samples. However, it must be stated, that in numerous cases no case history or information on prior treatment was available. When tissue samples were received, also immunostaining was performed demonstrating *Acanthamoeba* trophozoites and also cysts within the infected tissue (Fig.4).

Concerning the geographical origin, 44.5% of all cases were from Vienna, 35.5% from Styria, 10% from Upper Austria, 4% from Lower Austria and 2% each from Carinthia, Salzburg and Vorarlberg. There was no case from Burgenland. This, however, only reflects in which province of Austria the patients lived at the time point of diagnosis. Moreover, although our lab is the reference lab for all of Austria, most samples are of course received from Vienna, where our institution is located, also, Vienna holds approximately 25% of the Austrian population. In two cases, the site of infection could clearly be traced back to outside of Austria, one patient had acquired his infection when hiking in the Andes and not being able to properly clean his CLs for several weeks, the other patient, also a CL wearer, had been travelling South-East Asia for several months and developed symptoms before his return.

**Figure 5:**
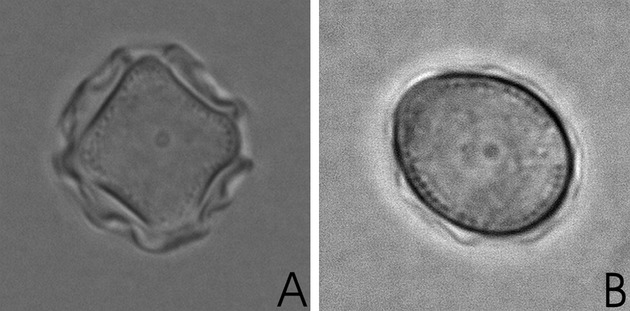
*Acanthamoeba* cysts. A. morphological group II: polygonal with wrinkled outer cyst wall (magnification: X1,000), B. Morphological group III: slightly smaller and rounded with ectocyst closely apposed to endocyst (magnification: X1,000).

Morphologically, the acanthamoebae found exhibited either a group II (Fig.5a) or a group III (Fig.5b) morphology, no representative of *Acanthamoeba* group I was isolated from any of the samples. The predominant genotype in the AK cases was T4, other genotypes found were T3, T5, T6, T10 and T11. All acanthamoebae isolated by culture were able to grow not only at 34 °C but also at 37 °C. In addition to acanthamoebae, in three cases *Vermamoeba vermiformis* was isolated, in all cases however, together with acanthamoebae and in none of the cases from a corneal scrape, but only from either CL cases or superficial corneal swabs. CL cases, if available, were usually heavily contaminated. In one CL case of a patient with severe AK, acanthamoebae were identified at a density of 10^5^/ml of lens case solution. Several CL cases were also contaminated with fungi, algae and nematodes.

### GAE cases

In cases of GAE, the age of the patients ranged from 2 to 25 yr (mean age 14.7), all GAE patients were HIV-negative, but two were severely immunosuppressed at the time of diagnosis. The first patient was a 25 yr old man from India, who had been living in Austria for 7 yr and had an underlying tuberculosis (Aichelburg et al. 2008), the second patient was a 17 yr old man from Austria who was immunocompetent and the amoebae most probably gained access to the CNS through a bone dehiscence of ethmoidal cells (Lackner et al. 2010) and the third patient was a 25 mo old boy with an underlying acute lymphoblastic leukaemia (Maritschnegg et al. 2011). The three GAE cases involved genotypes T2 (Walochnik et al. 2008), T5 and T4. Only in one case, patient no. 2, living acanthamoebae could be isolated from the CSF, however, all three patients already had a relatively long case history and had already been treated with various antimicrobial drugs. All three patients survived, but in all three cases it took several months until the patients recovered.

## Discussion

### Diagnostics

The direct detection of the causative agent in the clinical sample is the only reliable diagnostic method for AK infections. Detection of acanthamoebae on CLs and inside of lens storage cases is confirmatory, but must not be used exclusively as the diagnostic approach, as acanthamoebae can – although usually in very low densities – also be detected in CL cases of many asymptomatic CL wearers. Plate culture is still the mainstay for laboratory detection of *Acanthamoeba*, but, depending on the type of sample and the case history, does certainly not show 100% sensitivity. AK can easily be confused with a herpes simplex virus or fungal keratitis, thus there is often a significant delay before diagnosis is made and many patients have already undergone weeks of antimicrobial treatment. In our study, in initial samples, the sensitivity of the culture method was rather good (∼85%), but it gradually declined the longer the patient had been under treatment. Other authors report sensitivities of below 60% (Tu et al. 2008; Yera et al. 2007). Page and Mathers (2013) reviewing 12 yr of AK diagnostics report that from 112 AK cases investigated by culture only 28% were found to be positive by culture. Currently, PCR is the method of choice for *Acanthamoeba* detection, however, there is no commercial kit available. Expert knowledge is necessary to run reliable and reproducible PCRs in routine diagnostics. To our opinion, the PCR protocol established by Schroeder et al. (2001) is one of the best diagnostic PCRs currently available and moreover, also allows for genotyping by DNA sequencing of the amplicon. When only formalin-fixed material is available or if high sensitivity is needed, modifications or the use of other protocols are indicated. In the past years, numerous labs including our own have set up real-time PCR protocols for *Acanthamoeba* detection, and many very good protocols have been published, e.g. the one by Qvarnstrom et al. (2006) for simultaneous detection of *Acanthamoeba* spp., *B. mandrillaris* and *Naegleria fowleri*, which is very well suited for clinical samples.

### *Acanthamoeba* keratitis

During the 20 yr period of this study, 154 cases of AK were seen at our institute representing approximately 10% of the patients with an atypical keratitis and almost all cases were unilateral, which has also been reported from other long-term studies (Carvalho et al. 2009). In the past years, we saw around 10 AK cases per year, which would correspond to 0.125/100,000 inhabitants, however, *Acanthamoeba* diagnostics is also established in several other laboratories in Austria. In the UK, an annual incidence of around 0.14/100,000 inhabitants was reported for the years 1992–1996 and of up to 0.126/100,000 in the following years but the authors noted marked regional variations (Radford et al. 1998, 2002). In the USA, a rising incidence was noted starting from 2003 (Acharya et al. 2007), a recent study reporting 10.3 new cases per million population per year for the Portland metropolitan area (Page and Mathers 2013). In that study, a clear peak of AK cases during the summer months was registered, while in our study cases were equally distributed over the entire year. However, this is probably very much dependent not only on the local climate conditions but also on the type of local tap water supply and local recreational waters. In Vienna, as in almost all parts of Austria, tap water is mountain spring water and has a temperature of below 10 °C during the entire year. *Acanthamoeba* densities in Austrian tap water are extremely low and also most Austrian lakes are rather cold and, as far as these data are available, all have very low *Acanthamoeba* densities (own, unpublished data). However—although usually at low densities—acanthamoebae can be found in almost any habitat and regular contact to them is inevitable, which is also reflected by the fact that almost 100% of the normal population have specific antibodies against acanthamoebae (Walochnik et al. 2001).

In our study, 89% of those patients, for which background information was available, were CL wearers. Austria has an estimated number of 250.000 CL wearers, which corresponds to > 6% of the ametropic and 3% of the total population (Statistik Austria). In a study at the Moorefield's Eye Hospital in London 96% of the patients with diagnosed AK were CL wearers (Bernauer et al. 1996). In the USA, between 64% and 85% of AK patients have been reported to be CL wearers (Page and Mathers 2013; Stehr-Green et al. 1989). To our opinion, the most important risk factor for AK in Austria is poor CL hygiene. Singular amoebae gain access to the lens case via tap water or the air and then grow to immense densities within the container over usually prolonged periods of improper CL and lens case hygiene. Cleaning of CLs is generally more problematic in soft lenses, however, also here a rub and rinse cleaning method is beneficial and it is even more important to regularly exchange lenses and lens cases and use an appropriate cleaning solution. Lens cases should be cleaned manually and air-dried, because otherwise a biofilm develops and this, in combination with the use of one-step CL solutions, which do not sufficiently kill acanthamoebae (Hiti et al. 2005; Larkin et al. 1990), leads to extremely high amoebal densities. Besides amoebae and bacteria, also fungi, algae and nematodes were found in such microbial lens case communities. In the USA, an outbreak of AK associated with the use of a particular CL solution was recorded in 2007 (Verani et al. 2009). We could not prove any connection to the use of a particular CL solution, but the vast majority of our cases were wearers of soft CLs using multipurpose solutions. There were only few cases in CL wearers using two-step systems and only very few cases in wearers of rigid CLs. In many cases, CL wearers were involved, who do not regularly but only occasionally (e.g. once a week for sports) wear their CLs and who were often unaware of proper CL hygiene. Today, CLs can be purchased on the internet and thus many CL wearers have never been instructed on CL hygiene. A recent development is the use of coloured “party lenses” that are often re-used after long periods of time and are occasionally even exchanged among friends. Nevertheless, we had also eight proven AK cases in non-CL wearers, but six of these patients were > 60 yr old and had an underlying eye problem. Illingworth and Cook (1998) estimated that 10–15% of AK cases occur in non-CL wearers. Cases in non-CL wearers are often a consequence of ocular trauma and subsequent exposure to contaminated water or soil, e.g. in agricultural workers, but also cases in non-CL wearers without any other known predisposition for infection have been described (Jain et al. 2013, Parrish and O'Day 1991; Sharma et al. 2000). Furthermore, it is speculated that the individual external ocular flora (providing a nutrient source for the acanthamoebae) plays a significant role in the establishment of an infection. Differences and also changes in the commensal bacteria populating the eyelid margin, the conjunctiva and the tear film have been demonstrated in asymptomatic CL wearers (Larkin and Easty 1990).

Interestingly, in our study, women and men were affected almost equally, compared to most previous studies in which a majority of patients were female. This might be related to increased CL usage among males over the past decade. The age group with the highest incidence were clearly the 21–30-yr old patients. In a recent Australian study, eight of 13 cases were male and the mean age was 32 yr (Ku et al. 2009). Verani et al. (2009) reported 221 patients with a median age of 29 yr (range 12–76), 64% being female.

As far as this information was available, treatment consisted of local application of polyhexamethylene biguanide (PHMB, 0.02%) or chlorhexidine (0.02%) combined with propamidine isethionate (Brolene® eyedrops, Sanofi, Vienna, Austria; 0.1%) or hexamidine isethionate (Desomedine® eyedrops, Bausch & Lomb, Zug, Switzerland; 0.1%) and if indicated also with antibiotic eye ointment, however, treatment schemes varied greatly. The severe progression of the disease in many cases—keratoplasty was necessary in 19% of all patients—once again highlights the need for more awareness of the disease, not only among clinicians but also among CL wearers. CL wearers very often search medical help late, because they are used to minor irritations in the eye and awareness of ocular infections is generally low. Treatment is still problematic and might be improved by new options and combinations (Müller et al. 2012; Polat et al. 2014; Visvesvara 2010).

The predominant genotype in the AK cases was T4, other genotypes found were T3, T5, T6, T10 and T11. Genotypes T5 and T10 correspond to morphological group III, while T3, T4, and T11 exhibit a group II morphology. T6, at least the strain we isolated, has a group II morphology, but clusters within the group III genotypes (Walochnik et al. 2000). All of these genotypes have already been found as causative agents of AK and T4 is the predominant genotype in AK worldwide (Booton et al. 2009; Nuprasert et al. 2010; Risler et al. 2013; Spanakos et al. 2006). From neighbouring countries of Austria data concerning genotypes of AK causing strains is scarce. In an Italian study, altogether 15 AK isolates were typed and all belonged to genotype T4 (Gatti et al. 2010). The first cases of AK in Slovakia and Czech Republic were reinvestigated by genotyping the preserved strains and three isolates were shown to be T4 and one each T3 and T15 (Nagyová et al. 2010). From Germany recently an AK case caused by T13 was reported (Grün et al. 2014). In the USA, Verani et al. (2009) screened 22 outbreak-related isolates and found 20 of them being genotype T4, and one environmental isolate each of T3 and T14. In a recent study from China, all 14 AK causing acanthamoebae investigated were identified as genotype T4 (Zhao et al. 2010).

### Granulomatous amoebic encephalitis

Altogether three *Acanthamoeba* GAE cases have been diagnosed in Austria until now. From neighbouring countries, one case has been reported from Germany (Petry et al. 2006), and one case of *Balamuthia* GAE from the Czech Republic (Kodet et al. 1998). We found three different genotypes, T2, T4 and T5. The German case, as the vast majority of GAE cases around the world was caused by genotype T4, also cases involving genotypes T1, T10 and T12 have been published (Booton et al. 2005). To the best of our knowledge, no other GAE cases involving genotypes T2 and T5 have been published, however, only few GAE strains have been genotyped altogether and both genotypes are known as causative agents of AK (e.g. Maghsood et al. 2005; Spanakos et al. 2006). Recently, a new genotype, T18, has been isolated from a patient with fatal GAE (Qvarnstrom et al. 2013). Interestingly, this strain has a group I morphology, and thus is the first clearly pathogenic *Acanthamoeba* strain of this group. Except for the T4 strain (group II), the genotypes linked to GAE in our study cluster within morphological group III, the group comprising also genotypes T10 and T12, both known to cause GAE (Booton et al. 2005).

Altogether, it was shown that the annual incidence of AK in Austria currently lies around 0.125 cases per 100,000 inhabitants and that almost 90% of cases occur in CL wearers. Males and females were affected equally and most cases were recorded in the 21–30-yr-old patients. Moreover, three cases of GAE have been recorded in Austria. The most prevalent genotype in *Acanthamoeba* infections in Austria was by far genotype T4, other genotypes found were T2, T3, T5, T6, T10 and T11.
